# Changes in student behaviors and policy opinion regarding E-cigarettes at a Kentucky University from 2014 to 2018

**DOI:** 10.1016/j.pmedr.2021.101364

**Published:** 2021-03-22

**Authors:** Jason W. Marion, Alina Strand, Elliott Baldridge

**Affiliations:** Eastern Kentucky University, Department of Environmental Health Science, 521 Lancaster Ave., 220 Dizney Building, Richmond, KY 40475, USA

**Keywords:** E-cigarette, Vaping, Tobacco, University, Tobacco-free, College, Undergraduate students, Prevalence, Appalachia

## Abstract

The behaviors and opinions regarding e-cigarette use and campus policies prohibiting vaping vary greatly among college students nationally. Kentucky is one of the four U.S. states with the highest tobacco use prevalence, and characterizing e-cigarette use, trends and policy opinions among Kentucky undergraduates may inform interventions. To characterize population-level differences in e-cigarette-related behaviors and policy opinions among undergraduates from 2014 to 2018, results from two cross-sectional surveys (2014 and 2018) from a public regional university in south-central Kentucky were analyzed. Students from randomly selected undergraduate general studies courses completed a 5-minute in-class survey. Data were obtained from 514 and 519 respondents in 2014 and 2018, respectively. Mean age did not differ (19.9 and 20.1 years; p = 0.41) nor did class rank (p = 0.30) by survey year. Chi-square analysis indicated previous 30-day e-cigarette use was higher in 2018 than 2014 (28% vs. 18%; p < 0.001), and current cigarette use was lower in 2018 than 2014 (13% vs. 25%; p < 0.001). When current smoking and recent e-cigarette use were combined as a use variable, there was no significant difference between 2018 (29%) and 2014 (30%). Fraternity/sorority affiliation, being under 22 years old, male gender, out-of-state residency, and having a smoking parent were associated with recent e-cigarette use in multivariable logit models. Support for the on-campus vaping prohibition was lower among 2018 respondents (68% approval) compared to 2014 respondents (74% approval), respectively (p = 0.022). Overall, these findings may inform policy, population-specific health communications, and future research.

## 1 Introduction

Kentucky has been identified as a “high cigarette/e-cigarette” U.S. state, ranking among the top four states for both the prevalence of current e-cigarette use and current cigarette use (El-Shahawy et al. 2019). Additionally, the highest U.S. lung and bronchus cancer mortality and incidence rates are in Kentucky, particularly in the Appalachian region (National Cancer Institute 2020). Accordingly, monitoring the prevalence and risk factors for e-cigarette use in this region can inform health policies while strengthening assessments of federal policies in all communities.

Characterizing regional differences in tobacco use prevalence among college students will remain valuable for evaluating policies aimed at curbing youth and young adult tobacco use, such as national “Tobacco 21” (T21) legislation as signed into U.S. law in December 2019 as policy enforcement and compliance will likely vary by states and communities (Dobbs et al., 2020, Muralidharan et al., 2019). In tobacco-producing Kentucky, T21 has majority support (58%); however, support is less than national estimates of 75% support (Ickes et al., 2019a, Gentzke et al., 2020). The American Lung Association (2020) ranks Kentucky among 13 states with the weakest statewide restrictions on smoking with respect to Kentucky’s smoke-free regulations. Alternatively, Kentucky is among 20 states with a C-grade from the U.S. Vaping Index, which ranks Kentucky e-cigarette retail regulations related to taxes, flavors, and online sales as being more restrictive than the 24 states with A-grades (Consumer Choice Center, 2020).

Monitoring trends among young adults, including Kentucky undergraduates (traditionally 18 – 24 years old) are particularly meaningful as 99% of adult smokers started smoking before 26 years of age, with nearly 20% starting between 18 and 26 years of age (Centers for Disease Control and Prevention, 2012). If these initiation findings for smoking remain true or are more pronounced with e-cigarette use (Roberts et al. 2020), then this type of research regarding undergraduates has merit for informing public health interventions.

The objectives of this study were: (1) to investigate a hypothesized higher e-cigarette prevalence in 2018 than 2014 among undergraduate students attending an Appalachian Kentucky university, (2) to identify characteristics associated with recent e-cigarette use in this population, and (3) to assess support for the campus tobacco-free/vape-free policy [enacted in July 2014].

## 2 Methods

### 2.1 Data collection

Cross-sectional data were collected using a repeated cross-sectional study design. Specifically, data collection occurred during two time periods using similar study protocols approved by the Institutional Review Board at Eastern Kentucky University (#15–075 and #2018–1170). Data collection occurred during October 2014 and during March and April 2018 using a convenience sample each year. For reducing volunteer biases associated with online surveys (Ebert et al. 2018), a pen and paper design was used similar to the National Youth Tobacco Survey and Seo et al. (2011). In both years, 33 instructors in large general education courses (>40 students) were contacted to maximize participation for producing generalizable results for the whole campus. In 2014, 10 of 12 respondent instructors agreed to in-class surveys, and in 2018, 11 of 13 respondent instructors agreed. Surveys were completed in under five minutes. Among students physically in-class to receive surveys, over 95% in every classroom agreed to participate in 2014 and 2018, with most classes having 100% participation.

### 2.2 Survey instrument and measures

The questionnaires included 33 items. To assess e-cigarette use, participants responded to two items, “Have you ever used any electronic cigarettes, e-cigarettes, and/or vaping products?” and “If used, during the past 30 days, how frequently did you use electronic cigarettes, e-cigarettes, and/or vaping products?” To assess smoking status, participants reported if they have ever used cigarettes, how many cigarettes they smoked on average per day, and whether or not they had smoked at least 100 cigarettes or cigars in a lifetime. Current cigarette use was defined as lifetime smoking of 100 or more cigarettes and reporting current use of 0.5 or more cigarettes on average per day. “Recent use of e-cigarette or cigarette” was defined as recent e-cigarette use (any use in past 30 days) and/or current cigarette use.

Demographic information was obtained commensurate with college health assessments for enabling comparisons with national undergraduate data and informing potential health communications (American College Health Association, 2019). Participants reported their gender, race, age, year in college, on-campus/off-campus residential status, membership in the U.S. Armed Forces, membership in a social fraternity or sorority, and state in which they have lived in longest. Current parental use of cigarettes or e-cigarettes was recorded along with familial history of cancer and asthma. Nine items assessed opinions regarding the safety and regulation of e-cigarettes, and four items assessed opinions regarding the campus tobacco-free/vape-free policy approved in July 2014. Students rated their agreement or disagreement about statements, including “Eastern Kentucky University’s tobacco-free campus policy will make me healthier” and “EKU’s tobacco-free campus policy should continue to prohibit e-cigarette products from being used.” Opinions were assessed on a four-point scale from strongly agree to strongly disagree. Dichotomous coding for agree and disagree was performed.

### 2.3 Data analysis

Data were analyzed with Stata 15. Measures of frequency were examined for ordinal variables. Measures of central tendency, histograms, and t-tests were used for examining the continuous age variable by study year. Age was also dichotomously coded (≤ 21 years and > 21 years). Chi-Square tests were performed to assess differences by study year for dichotomous variables. Simple and multivariable logistic regression were used for examining associations between participant characteristics and/or behaviors with recent e-cigarette use. All logistic regression analyses were performed accounting for the cluster survey design since data were collected from individual classrooms (primary sampling units). The svyset and svy commands in Stata only accounted for the cluster design effect. The crude and adjusted odds ratio estimates were not changed (and remain unweighted); however, the 95% confidence intervals and *p*-values are larger given the non-random selection of study participants (Kreuter and Valliant, 2007). Final regression models (models 2 and 4) were made using the backward elimination method to predict the likelihood of e-cigarette usage in this population (Hosmer et al. 2013). Model fit was evaluated using the Hosmer-Lemeshow goodness-of-fit test and model discrimination was evaluated using the area under the ROC curve (AUC) (Hosmer et al. 2013).

## 3 Results and discussion

### 3.1 Participant demographics

Data were collected from 520 and 526 students in 2014 and 2018, respectively. Complete data were obtained from 489 (94%) in 2014 and 472 (90%) in 2018. No significant differences were observed by survey year in age (≤ 21 years vs. > 21 years), Kentucky residency, off-campus residency, participation in fraternity/sorority life, military service, family history of cancer or asthma, or parental history of smoking and/or vaping (p > 0.05; Table 1). There was no difference in mean participant age by year (p = 0.406) with means of 19.9 and 20.1 in 2014 and 2018, respectively. A greater proportion of women participated in 2014 (66%) than 2018 (53%). There were significantly less Hispanic and non-white participants in 2018 (3%) than 2014 (12%).

**Table 1:** Participant characteristics by study year (2014 and 2018) and crude odds ratios for recent^a^ e-cigarette usage at a regional university in Appalachian Kentucky.

Characteristic and/or Covariate	n (% of study pop.)			E-cigarette Use cOR (95% CI)^b^	
	2014	2018	*Χ^2^p*	2014 cOR	2018 cOR
Ever Used E-cigarette (Vaped)	173 (33)	241 (46)	<0.001	^c^	^c^
Recent^a^ E-cigarette Use	93 (18)	141 (28)	<0.001	^c^	^c^
One E-cigarette use per day	62 (12)	76 (15)	0.187	^c^	^c^
2 – 3 E-cigarette uses per day	12 (2.3)	28 (5.6)	0.009	^c^	^c^
4 – 6 E-cigarette uses per day	9 (1.8)	13 (2.6)	0.381	^c^	^c^
>7 E-cigarette uses per day	10 (2.0)	36 (7.2)	<0.001	^c^	^c^
Don’t know # E-cig uses per day	10 (2.0)	12 (2.4)	0.656	^c^	^c^
Recent^a^ Use of E-Cig or Cigarette	152 (30)	143 (29)	0.546	^c^	^c^
Recent Use of E-Cig & Cigarette	59 (12)	48 (10)	0.290	^c^	^c^
Current^d^ Cigarette Use	126 (25)	63 (13)	<0.001	***13 (7.4***–***22)***	***15 (7.7***–***28)***
< 21 years of age	449 (87)	447 (86)	0.561	1.2 (0.6–2.5)	***1.9 (1.0***–***3.6)***
Female gender	339 (66)	276 (53)	<0.001	***0.4 (0.2–0.6)***	***0.5 (0.4–0.8)***
White, non-Hispanic	456 (88)	453 (97)	<0.001	0.8 (0.4–1.5)	0.7 (0.3–2.2)
Kentucky Resident	426 (83)	424 (84)	0.695	0.8 (0.4–1.4)	***0.4 (0.3–0.7)***
Commuter Student	191 (37)	195 (38)	0.872	0.7 (0.4–1.2)	0.9 (0.6–1.3)
Fraternity/Sorority Life	99 (19)	103 (20)	0.707	1.5 (0.9–2.6)	***3.4 (2.1***–***5.4)***
Military Service	16 (3.1)	27 (5.3)	0.082	***3.1 (1.1***–***9.0)***	0.5 (0.2–1.4)
Any Cancer in Family	241 (46)	227 (44)	0.446	0.9 (0.6–1.4)	0.9 (0.6–1.3)
Any Asthma in Family	153 (29)	144 (28)	0.603	1.0 (0.6–1.6)	0.8 (0.5–1.2)
Parent(s) Smoke and/or Vape	194 (38)	184 (36)	0.430	***1.7 (1.1***–***2.7)***	1.1 (0.7–1.6)
Believe Policy Promotes Health	346 (67)	351 (67)	0.982	***0.3 (0.2***–***0.4)***	***0.3 (0.2***–***0.4)***
Support E-Cig Campus Ban	386 (74)	356 (68)	0.022	***0.3 (0.2***–***0.5)***	***0.2 (0.1***–***0.3)***

a: Recent: Anytime in the previous 30 days.

b: Crude odds ratios (cOR) and 95% confidence interval.

c: Not determined since the characteristic is or includes the outcome of the logistic regression.

d: Current: Smoked > 100 cigarettes in lifetime and currently average smoking more than zero cigarettes per day.

### 3.2 Prevalence of e-cigarette and cigarette use by study year

Daily, recent (30-day), and ever e-cigarette use increased significantly (p < 0.001) from 5.8%, 18%, and 33% in 2014 to 12%, 28%, and 46% in 2018, respectively (Table 1). Current cigarette use was significantly (p < 0.001) lower in 2018 (13%) than in 2014 (25%). The 2018 results are similar to 2018 results from a large Midwestern university proximal to Appalachia that reported 27.7% prevalence in past 30-day e-cigarette use among 2018 undergraduates (Roberts et al. 2020). Nationally, e-cigarettes are the most commonly used tobacco product among undergraduates; however, the U.S. undergraduate prevalence of self-reported 30-day e-cigarette use (15.2%) and cigarette use (7.5%) are appreciably lower than observed in this study or Roberts et al. (2020) (American College Health Association, 2019). Some differences from national data may be attributed to younger participant age (median age = 19 years) in general education courses, whereby younger age is associated with e-cigarette use (Table 2).

**Table 2:** Multivariable logistic regression models for recent^a^ e-cigarette use, with and without the current^b^ cigarette use covariate.

	Includes Cigarette Use		Cigarette Use Variable Excluded	
	Model 1	Model 2^c^	Model 3	Model 4^c^
Covariate	aOR^d^ (95% CI)	aOR^d^ (95% CI)	aOR^b^ (95% CI)	aOR^b^ (95% CI)
Year 2018 vs. 2014	***3.1 (1.9 – 5.3)***	***3.3 (1.1 – 2.4)***	***1.6 (1.1 – 2.3)***	**1.6 (1.1 – 2.4)**
≤ 21 years of age	2.2 (0.9 – 5.2)	*2.5 (1.1 – 5.6)*	1.7 (1.0 – 3.2)	***1.9 (1.2 – 3.0)***
Female Participant	***0.6 (0.4 – 0.8)***	**0.6 (0.4 – 0.9)**	***0.4 (0.3 – 0.6)***	***0.4 (0.3 – 0.6)***
Kentucky Resident	*0.5 (0.3 – 1.0)*	*0.5 (0.3 – 0.9)*	*0.5 (0.3 – 0.9)*	**0.5 (0.3 – 0.9)**
Fraternity/Sorority History	***3.1 (2.0 – 4.7)***	***3.1 (2.1 – 4.5)***	***2.3 (1.6 – 3.3)***	***2.5 (1.8 – 3.4)***
Parent(s) Smoke	1.5 (0.9 – 2.5)		1.5 (0.9 – 2.3)	*1.6 (1.0 – 2.4)*
Current^b^ Cigarette Use	***15 (8.1 – 27)***	***16 (8.6 – 28)***	–	–
White, Non-Hispanic	0.8 (0.5 – 1.4)	–	0.8 (0.6 – 1.2)	–
Active or Former Military	0.6 (0.3 – 1.5)	–	1.0 (0.4 – 2.6)	–
Commuter Student	1.0 (0.6 – 1.5)	–	0.9 (0.7 – 1.3)	–

a: Recent: Any e-cigarette use in the past 30 days.

b: Current: Smoked at least 100 cigarettes in lifetime and currently smokes more than zero cigarettes per day.

c: Model 2 and Model 4: Most parsimonious model following backwards stepwise regression.

d: Adjusted odds ratios (aOR) and 95% confidence intervals for covariates.

* Italics: *p* < 0.05

**Bold: *p* < 0.01;

***Bold and italics: *p* < 0.001.

Research characterizing ever e-cigarette use among Appalachian undergraduates remains limited. One Appalachian university study (n = 498) in 2018 reported 43% prevalence (Omoike and Johnson, 2021). which is similar to 46% reporting ever using e-cigarettes or vaping products (Table 1). These values are higher than the 2018 national prevalence (25.4%) of ever use among undergraduates (American College Health Association, 2019).

Among recent e-cigarette users, the majority (>50%) reported using e-cigarettes once per day or less in 2014 and 2018. The prevalence of e-cigarette use was significantly higher (p < 0.01) in 2018 than 2014 in two frequency categories (Table 1), including seven or more uses per day, which was 3.6-times higher in 2018 (6.8%) than in 2014 (1.9%).

### 3.3 Predictors of recent e-cigarette use

Current cigarette use, being 21 years of age or younger, male gender, participation in fraternity/sorority life, and having parents who smoke were all risk factors for recent e-cigarette use. Among multivariable models (Table 2), the saturated (Model 1) and parsimonious (Model 2) models including current cigarette usage had better discrimination in classifying recent e-cigarette users (Area under ROC curve [AUC] = 82.2 – 82.4%) than the two models excluding the cigarette term (AUC = 67.8 – 68.2%). Models 1, 2, and 4 had acceptable fit (p = 0.520, p = 0.151, and p = 0.208). Model 3 exhibited poor fit (p = 0.033). Multicollinearity was not observed as all VIFs were less than 2.

Closer examination at the fraternity/sorority life data showed that in 2014 and 2018, 47% and 63% of fraternity men were recent e-cigarette users compared to 24% and 25% in the non-fraternity male population, respectively. Fraternity men represented 11% of the 2018 participants, but represented 36% of the most frequent e-cigarette user group (>7 uses/day). No sorority members reported seven or more uses per day in either year. Among sorority women, recent e-cigarette use was higher (32% vs. 11%) in 2018 compared to 2014. These observations correspond with national data whereby fraternity/sorority members were twice as likely in 2017 to have recently used e-cigarettes than other undergraduates (Soule et al., 2019).

Among out-of-state undergraduates, recent e-cigarette use prevalence was two times higher in 2018 (44%) than 2014 (22%), and in both years, higher than the general population, resulting in lower odds of e-cigarette use by Kentucky residents (Table 2). Out-of-state residency in this study may be indicative of higher socioeconomic status relative to the general population as out-of-state tuition was considerably higher than in-state. If so, observed differences may be linked to socioeconomic status (SES), which has been observed with respect to Juul use at a Midwestern university (Roberts et al., 2020).

### 3.4 E-cigarette policy opinions

In both years, 67% agreed that the campus tobacco-free policy makes them healthier. Significantly less support for the campus e-cigarette use prohibition was observed in 2018 (68%) versus 2014 (74%) (p = 0.022), corresponding with a higher prevalence of e-cigarette usage in 2018. Stratified multivariable logistic regression analysis for predicting policy opposition determined that increased policy opposition in 2018 was confounded by recent e-cigarette use. Specifically, greater opposition to the policy prohibiting e-cigarette use was observed in 2018, but only in the e-cigarette user model (aOR = 1.82, [95% CI: 1.01 – 3.27]), and not in the non-user model (aOR = 1.04, [95% CI: 0.71 – 1.52]).

### 3.5 Study implications

The relatively high frequency of e-cigarette use in this mostly young adult population supports regulation (T21), educational campaigns, and further research. Educational campaigns are recommended at this university. Targeted education would be enhanced by research elucidating determinants of e-cigarette use (and initiation) among undergraduates. SES and other factors (e.g. cost-related decisions, attractiveness [Lee et al. 2017]), were not assessed, but some risk factors (fraternity/sorority life membership and out-of-state classification) may have been SES-related. Juul use, which was not directly assessed, but popular in 2018 (Kavuluru et al., 2019), has been linked to higher SES (Roberts et al. 2020). Inclusion of Juul in future surveys is recommended since 72% of Juul users in Ickes et al. (2019b) did not report e-cigarette use. Interventions in university settings could benefit from research assessing relationships between e-cigarette use and SES within fraternity and sorority populations. Interventions also could be enhanced if greater understanding existed on the role of peer influence on e-cigarette use in university settings as previous research described e-cigarette use as more socially acceptable than traditional cigarettes at a Midwestern university (Lee et al. (2017).

This report is specific to a predominantly white Appalachian regional comprehensive university and presumably included substantial representation of low-income students as 49% of students receive income-based Pell grant support according to 2018 university data. Misclassification from self-reported data may exist. These data compare different academic terms (Fall 2014 vs. Spring 2018) which presents a limitation. Overall, these findings demonstrate continued efforts aimed at preventing tobacco use among undergraduates attending Appalachian and Kentucky universities remain needed.

## CRediT authorship contribution statement

**Jason W. Marion:** Conceptualization, Methodology, Formal analysis, Investigation, Data curation, Writing - original draft, Writing - review & editing, Supervision, Project administration. **Alina Strand:** Conceptualization, Methodology, Investigation, Writing - original draft. **Elliott Baldridge:** Methodology, Investigation, Validation, Formal analysis, Writing - original draft.

## Declaration of Competing Interest

The authors declare that they have no known competing financial interests or personal relationships that could have appeared to influence the work reported in this paper.
